# The Genus *Cecropia*: A Biological Clock to Estimate the Age of Recently Disturbed Areas in the Neotropics

**DOI:** 10.1371/journal.pone.0042643

**Published:** 2012-08-10

**Authors:** Paul-Camilo Zalamea, Patrick Heuret, Carolina Sarmiento, Manuel Rodríguez, Anne Berthouly, Stéphane Guitet, Eric Nicolini, César Delnatte, Daniel Barthélémy, Pablo R. Stevenson

**Affiliations:** 1 IRD, UMR AMAP (Botanique et bioinformatique de l'architecture des plantes), Montpellier, France; 2 Departamento de Ciencias Biológicas, Universidad de Los Andes, Bogotá, Colombia; 3 INRA, UMR ECOFOG (Écologie des Forêts de Guyane), Kourou, French Guiana; 4 Museum d'Histoire Naturelle d'Aix-en-Provence, Aix-en-Provence, France; 5 CIRAD, UMR AMAP (Botanique et bioinformatique de l'architecture des plantes), Montpellier, France; 6 Office National des Forêts, DTRD, Cayenne, French Guiana; 7 INRA, UMR AMAP (Botanique et bioinformatique de l'architecture des plantes), Montpellier, France; 8 CIRAD, BIOS Direction, Montpellier, France; DOE Pacific Northwest National Laboratory, United States of America

## Abstract

Forest successional processes following disturbance take decades to play out, even in tropical forests. Nonetheless, records of vegetation change in this ecosystem are scarce, increasing the importance of the chronosequence approach to study forest recovery. However, this approach requires accurate dating of secondary forests, which until now was a difficult and/or expensive task. *Cecropia* is a widespread and abundant pioneer tree genus of the Neotropics. Here we propose and validate a rapid and straightforward method to estimate the age of secondary forest patches based on morphological observations of *Cecropia* trees. We found that *Cecropia*-inferred ages were highly correlated with known ages of the forest. We also demonstrate that *Cecropia* can be used to accurately date disturbances and propose twenty-one species distributed all over the geographical range of the genus as potential secondary forest chronometer species. Our method is limited in applicability by the maximal longevity of *Cecropia* individuals. Although the oldest chronosequence used in this study was 20 years old, we argue that at least for the first four decades after disturbance, the method described in this study provides very accurate estimations of secondary forest ages. The age of pioneer trees provides not only information needed to calculate the recovery of carbon stocks that would help to improve forest management, but also provides information needed to characterize the initial floristic composition and the rates of species remigration into secondary forest. Our contribution shows how successional studies can be reliably and inexpensively extended without the need to obtain forest ages based on expensive or potentially inaccurate data across the Neotropics.

## Introduction

Neotropical forests are the greatest terrestrial reservoirs of biodiversity and carbon [1]. Despite prolonged attention of researchers to habitat degradation, forest fragmentation and deforestation [2], currently tropical forests are the most threatened ecosystems worldwide [3]. High rates of deforestation and selective logging in turn have resulted in a large increase in secondary forest areas [4]. The resilience of secondary forests is poorly understood, although Norden *et al.* [5] showed that secondary forests could serve as habitat refugia and biodiversity reservoirs of tree species from mature tropical forests.

A common problem with studies of secondary forest ecology and succession, however, has been uncertainty over stand age. Tropical secondary forest succession is a process that takes several decades, and to our knowledge, no continuous data on vegetation change are available for secondary forests over periods longer than 30 years. Consequently, the chronosequence approach, where disturbance ages of secondary forests are known, has been frequently used to study secondary forest dynamics [6]. Nevertheless, due to the difficulty of assessing the ages of secondary forest areas [7], there is a clear need for accurate and repeatable methodologies that allow us to date the age of disturbances over decadal time scales. More recently, attention has also been given to the need to monitoring and quantification of carbon sequestration processes to implement mechanisms for Reducing Emissions from Deforestation and Forest Degradation (REDD). However, one of the critical limitations in doing so is accurately estimating forests ages [8].

Currently, the most frequently used methods to date secondary forest are interviews of local people, use of remote-sensing techniques, probabilistic approaches based on tree growth rates, ^14^C dating, and tree ring analysis [7], [9]–[10]. All of these methods have limitations [9]. For example, information from local people is not always available, and its accuracy is highly variable [7]. Remote sensing or ^14^C dating are inaccurate and/or expensive techniques that may only work when suitable material is available.

Here, we demonstrate that *Cecropia*, a widespread and abundant pioneer genus in the Neotropics, is an accurate chronometer for dating different types of disturbance. We base this demonstration on a causal chain of previous observations, i.e. the *Cecropia* developmental periodicity allows predicting the age of individuals of this genus [11]–[12]. In secondary forests, the age of individuals of pioneer tree species reflects age of the forest and thereby age of the disturbance, since these species are incapable of germination following canopy closure [7]. In this study, we tested whether our estimation of *Cecropia* age based on two species and three different types of disturbance was reliable to the age of disturbance by studying the relationship between the estimated age of *Cecropia* and the real age of the disturbance. In addition, we propose twenty-one *Cecropia* species distributed all over the geographical range of the genus as potential chronometer species.

## Materials and Methods

### Ethics statement

Procedures and permits for measuring living *Cecropia* trees for the slash-and-burn agricultural sites in Colombia and French Guiana were approved and obtained from private local owners on each locality. Procedures and permits for measuring and handling living *Cecropia* trees on the gold mining sites and the forestry road were approved and obtained from the French Guiana ONF (*Office National des Forêts*), which is in charge of building the logging road-network and controlling the environment on the mining sites.

### Study species

In this study, we focused on two *Cecropia* species characterized by different geographic distributions. *C. sciadophylla* is a widespread species distributed throughout the Amazon basin, the Llanos region of Colombia and Venezuela, and the Guiana shield, while *C. obtusa* has a limited distribution on the Guiana shield and lower Amazon basin [13].

### Study area

We gathered data for three different types of disturbance: i) slash and burn agriculture, ii) a forestry road, and iii) gold mining sites. For the slash-and-burn agricultural sites, stand ages were obtained through interviews with local people, while for the forestry road and the mining sites, ages were obtained via the French Guiana ONF databases, ensuring the accuracy of the disturbance age. For the slash-and-burn chronosequences, data were gathered in three different sites, two in Colombia and one in French Guiana. i) The first Colombian chronosequence was located ∼11 km from Leticia, in an agricultural matrix composed of mature forest, secondary forest, and native crop stands (*Huitoto* native community), located in the department of Amazonas (4°6′S, 69°57′W). The Leticia chronosequence was composed of 8 age classes between 2 and 18 yrs. old. ii) The second Colombian chronosequence was located at the municipality of La Primavera, a natural savanna located in the department of Vichada (5°24′N, 69°53′W); the landscape matrix was composed by savanna vegetation and gallery forest, where some years ago traditional slash-and-burn agricultural practices were developed. The stands established at La Primavera were composed of 5 age classes between 5 and 20 yrs. old. iii) The French Guiana chronosequence was located at Sparwine town in an agricultural matrix composed of mature forest, secondary forest, and native crop stands (near the border between French Guiana and Suriname, 5°16′N, 54°14′W). The Sparwine chronosequence was composed of 9 age classes between 1 and 14 yrs. old.

Data on the forestry road and mining disturbances were also gathered in French Guiana. The Counamama 60 km-long forestry road is located between the municipalities of Sinnamary and Iracoubo (5°18′N, 53°13′W). This road was constructed as an access way for a logging concession. Its construction started in 1989 and finished in 2006; during the construction process, different sections were opened successively generating a chronosequence composed of nine age classes that varied between 1 and 17 yrs. old. The Coralie gold mining sites were located between the municipalities of Roura and Regina in French Guiana (4°29′N, 52°27′W). Six sites corresponding to 6 different age classes that varied between two and eight years old were chosen. In order to avoid any bias generated by the prior knowledge of stand ages, the real stand ages were always obtained after the estimation of *Cecropia* age.

### Plant material and measurements

In the French Guianan localities a total of 122 individuals of *C. sciadophylla* and 287 individuals of *C. obtusa* were measured, while in the Colombian localities a total of 80 individuals of *C. sciadophylla* were measured. Only straight trees without any evident trauma were selected. Although *C. sciadophylla* and *C. obtusa* are the most abundant *Cecropia* species in French Guiana, the proportion of the two species is not the same in all sites. The abundance of *C. sciadophylla* individuals relative to *C. obtusa* individuals is lower in mining and slash-and-burn agricultural sites.

**Figure 1:**
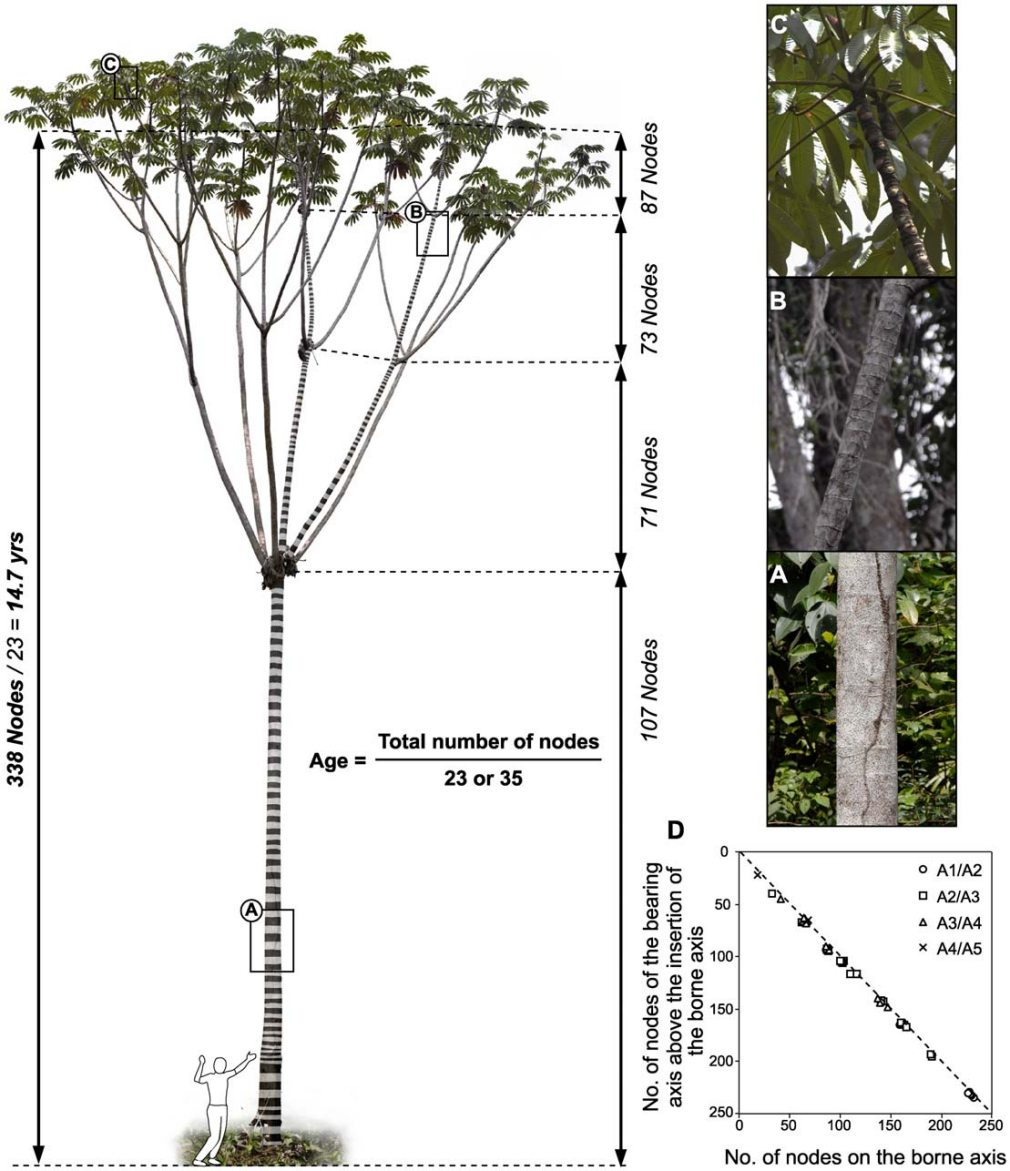
Tree silhouette showing a 14.7-years-old individual of *C. sciadophylla*. *Cecropia* age estimation protocol consists in dividing the total number of nodes on the main axis by 23 for *C. sciadophylla* or 35 for *C. obtusa*. In the pictures (a), (b), and (c) leaf and stipule scars are shown at different heights in the tree. (d) Relationship between the number of nodes on the borne axes (An) and that of their bearing axis (An-1) above their point of insertion, for the same individual. The comparison of A2 in relation to A1 is represented by circles, A3 in relation to A2 by squares, A4 in relation to A3 by triangles and A5 in relation to A4 by “x” symbols. The dotted line in the panel (d) represents the 1∶1 line. The human silhouette represents a 1.8 m height scale.

Previous studies of two species, *C. obtusa* and *C. sciadophylla*, showed a high annual periodicity in reproductive and branching processes, as well as an annual alternation of long and short nodes [11]–[12]. Throughout plant ontogeny and over a wide geographic gradient, our previous results show that the production of ∼35 and ∼23 nodes per year is a remarkably stable trait for *C. obtusa* and *C. sciadophylla*, respectively. These results have shown that the ages of individual trees can be estimated through observations of external morphology. This occurs because the scars of fallen leaves, inflorescences and branches remain visible along the trunk in *Cecropia* species (Fig. 1) [11]–[12]. In this study we will use our previous results as a background to test the hypothesis that *Cecropia* could be used as a biological clock to estimate the age of recently disturbed areas.

The protocol that we propose for estimating the age of *Cecropia* individuals is thus based on morphological description of the main axis from the base to the apex (Fig. 1). The protocol consists in dividing the total number of nodes on the main axis by 23 for *C. sciadophylla* or 35 for *C. obtusa* (Fig. 1). When trees are higher than 2–3 m the use of binoculars is necessary to count the number of nodes. In order to determine the accuracy of the counting of nodes using binoculars, the total number of nodes was counted in 91 individuals (56 *C. obtusa* and 35 *C. sciadophylla*) at the Counamama forestry road using binoculars, and then those individuals were felled and the total number of nodes was manually re-counted. Regardless of the *Cecropia* species, we found that the total number of nodes counted using binoculars was highly correlated with the actual total number of nodes (regression slope = 0.962, intercept = 0.172, R^2^ = 0.983, P<0.001). In addition, we observed that for *C. obtusa* and *C. sciadophylla* individuals, the number of nodes per axis was positively correlated with the number of nodes of the bearing axis above its insertion point, suggesting that the emission of new nodes occurs at the same rate in all axes of a given individual ([11], [14]; see Fig. 1d). Thus, for dating purposes, if the main axis is dead or broken, it is possible to estimate the age by following the main axis and then counting nodes on a vigorous branch.

### Generality

Given that annual flowering is one of the traits used to estimate the age of *C. obtusa* and *C. sciadophylla* individuals [11]–[12], it is crucial to determine whether our chronometric approach is likely to be broadly applicable across *Cecropia* species. Zalamea *et al.* [15] used a herbarium dataset to characterize the reproductive phenology of 35 *Cecropia* species, and found that 21 of them are characterized by an annual flowering. Here we used this information, to generate the geographic distribution map of species presence. To do that, we divided the area comprised from 24°N to 31°S and from 104°W to 34°W into hexagons of 4° side, and then, based on 2244 herbarium vouchers, we mapped the presence of annually flowering species by hexagons using ArcMap [16].

### Data analysis

The relationship between the estimated age of *Cecropia* and the real age of disturbances was assessed using linear-mixed effect models, including the locality of each tree as a random factor. We also included the following covariates as fixed effects: species, disturbance type, interaction between the real age of the disturbance and *Cecropia* species, and interaction between the real age of the disturbance and disturbance type. To determine a confidence interval for the correlation coefficient between the estimated *Cecropia* age and the real age of the disturbance, we performed a Pearson correlation analysis between paired samples, and used a bootstrap procedure with 10000 replicates. *Cecropia* estimated age and real age of the disturbance were both square-root transformed to meet normality and homoscedasticity assumptions. We used restricted maximum likelihood estimation in the mixed-effect model, and two-tailed tests with a significance level set to α = 0.05. All analyses were made in R software [version 2.13.1; 17] using the nlme [version 3.1; 18] package.

## Results

**Figure 2:**
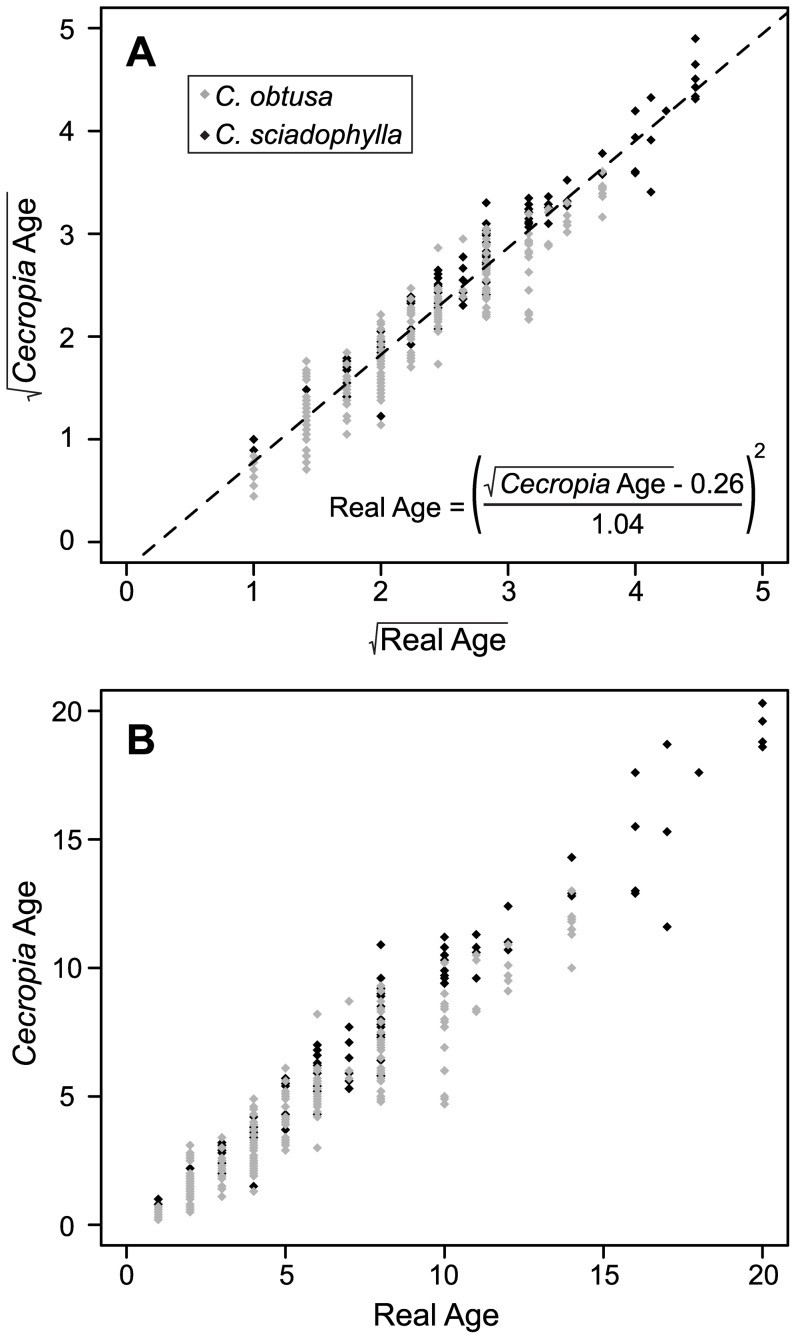
Relationship between the estimated age of *Cecropia* trees obtained using the age estimation protocol (see **Fig. 1**) and real age of disturbances determined using multiple datasets from local interviews and ONF information. (a) Both ages were square-root-transformed to meet normality and homoscedasticity assumptions. The dotted line represents the equation adjusted by the linear mixed-effect model. For ease of interpretation by the reader we also present the untransformed data in the panel (b). Black diamonds represent *C. sciadophylla* and grey diamonds represent *C. obtusa* individuals. For the Colombian sites we described 52 *C. sciadophylla* individuals at Leticia, while 28 were described at La Primavera. For the French Guiana sites, we described 20 *C. sciadophylla* and 137 *C. obtusa* individuals at Sparwine, 84 *C. sciadophylla* and 82 *C. obtusa* individuals at Counamama, and 18 *C. sciadophylla* and 68 *C. obtusa* individuals at Coralie (see the methods section for a detailed study site description).

We found that *Cecropia* estimated age was highly correlated with the disturbance real age (F_1,31_ = 701.8; P<0.001; Pearson's correlation: r^2^ = 0.97, P<0.001; Fig. 2a), the slope is not different from 1 (slope = 1.04), and the intercept is different from zero (intercept = −0.26). This relationship was neither different between the two *Cecropia* species nor among the disturbance types (interaction P values≥0.152). Although *Cecropia* estimated age and real age of the disturbance were both square-root transformed to meet normality and homoscedasticity assumptions, we also present untransformed data in figure 2b. To determine a confidence interval for the correlation coefficient between estimated *Cecropia* age and disturbance real age we performed a non-parametric bootstrap. The size of the confidence interval was very small (95% CI: 0.965–0.976), indicating that the uncertainty in our correlation coefficient is small.

**Figure 3:**
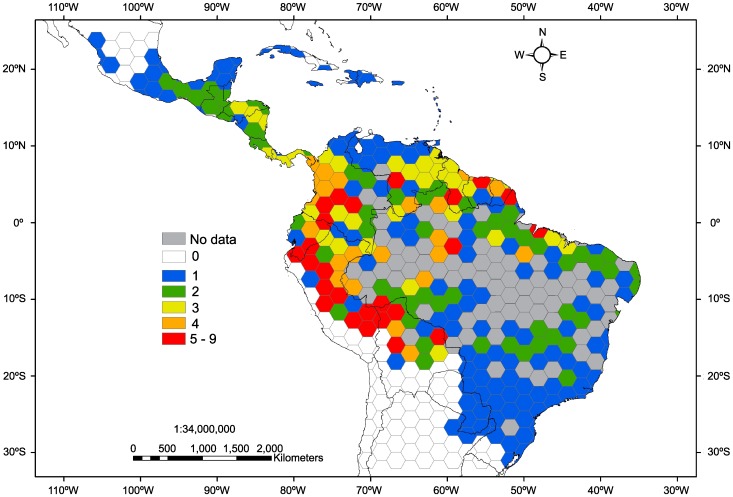
Geographic distributions of the annually flowering *Cecropia* species. The map illustrates the number of annually flowering *Cecropia* species identified by Zalamea et al. [15]. The colors represent the number of species present in each hexagon of 4° side (see Table 1 for a list of species).

Based on the presence of 21 *Cecropia* species that show annual flowering [15], we generated a distribution map to highlight some of the potential *Cecropia* chronometers available across the Neotropics (Fig. 3 and Table 1).

**Table 1:** List of *Cecropia* annual flowering species that could be used to date perturbations in the Neotropics.

Species	Distribution	Altitude (masl)	Range	Country presence*
*C. concolor*	Lower and central Amazon basin	0–600	Intermediate	Bol, Bra, Per
*C. distachya*	Throughout the Amazon basin	0–600	Wide	Bol, Bra, Col, Ecu, Fgu, Per, Ven
*C. engleriana*	Upper Amazon basin	0–700	Intermediate	Bol, Bra, Col, Ecu, Per, Ven
*C. insignis*	From Honduras to the Pacific coast of Ecuador	0–1400	Wide	Col, Cos, Ecu, Hon, Nic, Pan
*C. latiloba*	Guiana shield, Amazon and Orinoco basins	0–400	Wide	Bra, Bol, Col, Ecu, Fgu, Guy, Per, Sur, Ven
*C. longipes*	Eastern Panama and Northwestern Colombia	0–300	Local	Col, Pan
*C. montana*	Eastern slopes and foothills of the Andes	400–1500	Intermediate	Col, Ecu, Per
*C. mutisiana*	Upper Magdalena valley	500–1800	Local	Col
*C. obtusa*	Guiana shield and lower Amazon basin	0–500	Intermediate	Bra, Fgu, Guy, Sur
*C. obtusifolia*	From Mexico to the Pacific coast of Ecuador	0–1650	Wide	Bel, Col, Cos, Ecu, Gua, Hon, Mex, Nic, Pan, Sal
*C. pachystachya*	Southern fringes of the Amazon basin to Northern Argentina	0–1200	Wide	Arg, Bra, Par
*C. palmata*	Guiana shield, lower and central Amazon basin	0–300	Wide	Bol, Bra, Fgu, Sur
*C. pastasana*	From Colombia to Peru	900–2300	Intermediate	Col, Ecu, Per
*C. peltata*	From Mexico o Northern fringes of the Amazon basin	0–2000	Wide	Bel, Bra, Col, Cos, Gua, Guy, Jam, Mex, Nic, Pan, Sur, Tri, Ven
*C. polystachya*	From Peru to Bolivia	0–1800	Intermediate	Bol, Bra, Per
*C. purpurascens*	Central Amazon basin, near to Manaus	0–300	Local	Bra
*C. saxatilis*	Central Brazil to eastern Bolivia	200–1400	Intermediate	Bol, Bra,
*C. schreberiana*	Western Indies	0–1100	Wide	Cro, Cub, Doi, Dom, Gud, Hai, Pue, Jam, Joh, Luc, Mar, Mon, Sab, Tor, Vin
*C. sciadophylla*	Guiana shield, Amazon and Orinoco basins	0–1300	Wide	Bol, Bra, Col, Ecu, Fgu, Guy, Per, Sur, Ven
*C. strigosa*	From Northern Peru to Bolivia	400–1900	Intermediate	Bol, Per
*C. utcubambana*	Southern Ecuador to Southern Peru	400–2200	Intermediate	Ecu, Per

The list was taken from [15]. For each species, the distribution, altitude, geographic range and country presences were taken from [13] and personal observations.

* Arg = Argentina, Bar = Barbados, Bel = Belize, Ber = Bermuda, Bol = Bolivia, Bra = Brazil, Col = Colombia, Cos = Costa Rica, Cub = Cuba, Dom = Dominica, Ecu = Ecuador, Sal = El Salvador, Fgu = French Guiana, Gud = Guadeloupe, Gua = Guatemala, Guy = Guyana, Hai = Haiti, Hon = Honduras, Jam = Jamaica, Mar = Martinique, Mex = Mexico, Mon = Monserrat, Net = Netherland Antilles, Nic = Nicaragua, Pan = Panama, Par = Paraguay, Per = Peru, Pue = Puerto Rico, Doi = Dominican Republic, Sab = Saba, Cro = St. Croix, Joh = St. John, Luc = St. Lucia, Vin = St. Vincent, Sur = Suriname, Tor = Trotola, Tri = Trinidad, and Ven = Venezuela.

## Discussion

The correlation coefficient of the relationship between *Cecropia* estimated age and disturbance real age was very close to one. Moreover, in a previous study Zalamea [14] showed that the production of new nodes is extraordinarily stable throughout plant ontogeny and among populations of *C. sciadophylla*, both within and among years. This stability was also observed despite a high variation in annual precipitation and a wide geographic range among the study sites, suggesting a strong genetic control and a weak climatic or environmental influence on the observed periodicity. Together, both results show the high potential of the genus *Cecropia* as a proxy to estimate the age of secondary forests in the Neotropics.

In the relationship between *Cecropia* estimated age and disturbance real age (Fig. 2), the intercept was negative and close to zero, suggesting a small lag in recruitment of *Cecropia* individuals after perturbation. In anthropogenic perturbations, such as slash-and-burn agriculture, mining exploitation, and road construction, soil is highly disturbed, and seed banks could be affected or eliminated. For example, fires lasting five hours, such as those commonly applied when clearing land for agriculture, are sufficient to eliminate the *C. sciadophylla* seed bank up to a depth of 15 cm (Zalamea & Stevenson unpublished data). *Cecropia* is common on disturbed areas affected by fire and mining, indicating that recruits may come from seed rain rather than soil seed bank. Thus, the small lag in recruitment could be a response by timing of disturbance relative to seed rain, yet this lag is no longer than a year. In Barro Colorado Island, Panama, most *C. insignis* germination occurred in natural gaps within the first year after gap opening [19], and in central Amazonia, *C. sciadophylla* and *C. purpurascens* form even-aged stands in gaps resulting from different land uses [20], suggesting that *Cecropia* recruitment occurs in a single pulse, early in the regeneration process. Although several factors could delay recruitment, the intercept close to zero show that this delay in our database, is short in time, usually a few months.

Our method is limited in applicability by the maximal longevity of *Cecropia* individuals. Assessing individual longevity in tropical trees is challenging [21]–[24], and in the case of *Cecropia* species, longevity data are scarce. In *Cecropia*, these estimations vary among 35 years for *C. obtusifolia* [9], 54 years for *C. sciadophylla* [25] and 96 years for *C. insignis* [26]. Although the oldest chronosequence used in this study was 20 years old, in a recent study we have dated a 34 years old *C. sciadophylla* individual in French Guiana [12]. Thus, at least for the first four decades after disturbance, the method described in this study provides very accurate estimations of secondary forest ages.

Although *Cecropia* is a widespread genus on the Neotropics, the method proposed here to estimate the age of disturbed areas could not be used yet for all the species of the genus. This is because we do not know how many nodes are produced per year for each *Cecropia* species. However, as flowering is one of the traits we used to estimate the age of *Cecropia* individuals in our previous studies [11]–[12], annual flowering periodicity suggests high potential for *Cecropia* species to become secondary forest chronometers, as it has been proposed for *C. obtusa* [11] and *C. sciadophylla* [12]. The distribution map of the annually flowering *Cecropia* species (Fig. 3) implies that the *Cecropia* dating method could be used across a wide geographical range (i.e. all the Neotropics). Preliminary results showed an annual growth periodicity for *C. angustifolia, C. ficifolia, C. insignis* and *C. distachya* (P.C. Zalamea and P. Heuret, unpublished data). These results reinforce the idea that *Cecropia* could be used as a chronometer to estimate the time since forest disturbance. Although this study was based in anthropogenic disturbances, this methodology could also be used to date natural gaps. However, the dense canopy occurring in natural gaps compared to anthropogenic disturbances complicates the *Cecropia* age estimation using binoculars (i.e. it is difficult to count all nodes along the main axis). In such cases, an accurate estimation could be made using a destructive method.

The age of pioneer trees not only provides information needed to calculate the recovery of carbon stocks that would help to improve forest management [8], but also provides information needed to characterize the initial floristic composition and the rates of species remigration into secondary forest [5], [7]. In addition, this information could be useful to establish differences in substrate or soil fertility among successional stands and to model secondary forest population dynamics [9], [22]. In the current Neotropical scenario of forest fragmentation and degradation, little is known about the variability of responses from different primary forest to degradation effects [5], [27]. Thus, our contribution shows how successional studies can be reliably and inexpensively extended without the need to obtain forest ages based on remote sensing data or potentially inaccurate interview data across the Neotropics. In addition, growth-monitoring studies in secondary forests in the Neotropics are scarce and recent, suggesting that integrative approaches that use chronosequences and permanent plots can provide the valuable knowledge urgently needed in the current portfolio of tools for secondary forests management.
